# White matter injury restoration after stem cell administration in subcortical ischemic stroke

**DOI:** 10.1186/s13287-015-0111-4

**Published:** 2015-06-19

**Authors:** Laura Otero-Ortega, María Gutiérrez-Fernández, Jaime Ramos-Cejudo, Berta Rodríguez-Frutos, Blanca Fuentes, Tomás Sobrino, Teresa Navarro Hernanz, Francisco Campos, Juan Antonio López, Sebastián Cerdán, Jesús Vázquez, Exuperio Díez-Tejedor

**Affiliations:** Department of Neurology and Stroke Center, Neuroscience and Cerebrovascular Research Laboratory, La Paz University Hospital, Neuroscience Area of IdiPAZ Health Research Institute, Autónoma University of Madrid, Paseo de la Castellana 261, 28046 Madrid, Spain; Department of Neurology, Clinical Neurosciences Research Laboratory, Hospital Clínico Universitario, Health Research Institute of Santiago de Compostela (IDIS), University of Santiago de Compostela, Travesía de Choupana, s/n, 15706 Santiago de Compostela, Spain; Laboratory for Imaging and Spectroscopy by Magnetic Resonance (LISMAR), Institute of Biomedical Research Alberto Sols, CSIC-UAM, Arturo Duperier, 4, 28029 Madrid, Spain; Cardiovascular Proteomics Laboratory & Proteomics Unit, Centro Nacional de Investigaciones Cardiovasculares, CNIC, Melchor Fernández, Almagro, 3, 28029 Madrid Spain

## Abstract

**Introduction:**

Despite its high incidence, nerve fiber (axon and myelin) damage after cerebral infarct has not yet been extensively investigated. The aim of this study was to investigate white matter repair after adipose-derived mesenchymal stem cell (ADMSC) administration in an experimental model of subcortical stroke. Furthermore, we aimed to analyze the ADMSC secretome and whether this could be implicated in this repair function.

**Methods:**

An animal model of subcortical ischemic stroke with white matter affectation was induced in rats by injection of endothelin-1. At 24 hours, 2 × 10^6^ ADMSC were administered intravenously to the treatment group. Functional evaluation, lesion size, fiber tract integrity, cell death, proliferation, white matter repair markers (Olig-2, NF, and MBP) and NogoA were all studied after sacrifice (7 days and 28 days). ADMSC migration and implantation in the brain as well as proteomics analysis and functions of the secretome were also analyzed.

**Results:**

Neither ADMSC migration nor implantation to the brain was observed after ADMSC administration. In contrast, ADMSC implantation was detected in peripheral organs. The treatment group showed a smaller functional deficit, smaller lesion area, less cell death, more oligodendrocyte proliferation, more white matter connectivity and higher amounts of myelin formation. The treated animals also showed higher levels of white matter-associated markers in the injured area than the control group. Proteomics analysis of the ADMSC secretome identified 2,416 proteins, not all of them previously described to be involved in brain plasticity.

**Conclusions:**

White matter integrity in subcortical stroke is in part restored by ADMSC treatment; this is mediated by repair molecular factors implicated in axonal sprouting, remyelination and oligodendrogenesis. These findings are associated with improved functional recovery after stroke.

**Electronic supplementary material:**

The online version of this article (doi:10.1186/s13287-015-0111-4) contains supplementary material, which is available to authorized users.

## Introduction

White matter injury and the mechanisms of nerve fiber (axon and myelin) repair have seldom been investigated in translational stroke research [1, 2], despite the fact that blood supply disruption also compromises whole axons and fibers and therefore brain connectivity. Even though relevant, white matter injury in stroke has not been extensively studied in the past due to the intrinsic difficulties associated with animal models; for instance, the fact that the rodent brain has substantially less white matter than higher mammals or humans [3, 4]. However, not only are up to 25 % of ischemic strokes in humans subcortical [1], but cortical infarcts also produce white matter injury. The high incidence of such damage motivates the search for an effective therapy that would enhance the mechanisms underlying the repair of damaged nerve fibers after any kind of stroke.

Stem cell therapy has demonstrated its efficacy in cortical stroke and may have a positive effect on subcortical lesions. In this regard, preclinical studies indicate that adipose-derived mesenchymal stem cells (ADMSC) are a promising new therapy for subcortical stroke that could promote recovery by improving the global brain repair mechanisms [5–7]. Trophic factor release, paracrine interactions and immunomodulatory effects have been suggested as the main functional mechanisms involved in ADMSC therapy [8, 9]. In this regard, stem cells are known to have paracrine effects on neurogenesis, gliogenesis, synaptogenesis, vasculogenesis and immunomodulation. However, there is little evidence whether stem cell administration can promote oligodendrogenesis and white matter fiber repair when axonal tract integrity has been compromised.

Therefore, the aim of this study was to investigate the therapeutic effects (improvement of functional deficits and enhancement of white matter fiber repair) of the intravenous administration of ADMSC in rats submitted to subcortical stroke with white matter injury.

## Methods

### Ethics statement

The procedure was carried out at our Cerebrovascular and Neuroscience Research Laboratory, La Paz University Hospital, Madrid, Spain. All experiments were designed to minimize animal suffering in compliance with, and approved by, our medical school’s Ethical Committee of La Paz University Hospital for the Care and Use of Animals in Research according to the Spanish and European Union rules (86/609/CEE, 2003/65/CE, 2010/63/EU, RD 1201/2005 and RD53/2013).

### Animals and surgery

A total of 72 male Sprague–Dawley rats weighing 200–250 g (Charles River Laboratories, France) were used. In all animals, the femoral artery was cannulated during surgery and induction of cerebral ischemia to allow continuous monitoring of physiological parameters including blood glucose levels, blood gases and blood pressure (Omicron ALTEA Monitor; RGB Medical Devices, Madrid, Spain). Cranial and body temperature were monitored and maintained at 36.5 ± 0.5 °C. Male Sprague–Dawley rats (200–250 g) were anesthetized using 3.5 % isoflurane in 2 L/minute oxygen and given meloxicam 2 mg/kg for analgesia. To provoke white matter injury, subcortical stroke was induced by injection of 1 μL endothelin-1 (ET-1; Calbiochem, Germany) (0.25 μg/μL) with the use of a SYR 5 μL Hamilton syringe (Tecknokroma, Barcelona, Spain) into the striatum using stereotactic references (+0.4 mm AP, +3.5 mm L, + 6 mm DV from bregma) as previously described [10].

After 24 hours, the treatment group received intravenously (i.v.) 2 × 10^6^ ADMSC in 1 ml of saline solution (n = 24) by the tail vein. Dose was determined based on previous studies [8, 9]. In the control (n = 24) and sham-operation (n = 24) groups, only saline solution was i.v. administered via the tail vein. Rats were sacrificed at 24 hours (n = 4 in each group for comparative anatomical analysis of the lesion in fresh tissue and to analyze ADMSC distribution), 7 days (n = 10 in each group) or 28 days (n = 10 in each group) after treatment for cell death and proliferation analysis and immunohistochemistry, immunofluorescence and Western blot studies.

### Cell culture protocol

ADMSC obtained from allogeneic adipose tissue of Sprague–Dawley rats (250–300 g) were cultured. The adipose tissue was digested with collagenase (Sigma Aldrich, Madrid, Spain) and incubated at 37 °C in 5 % CO_2_. On the third pass, the cell cultures were divided into three groups: 1) 1.0 × 10^5^ ADMSC for characterization, 2) 1.5 × 10^6^ ADMSC for proteomics analysis of the culture supernatant, and 3) 42 × 10^6^ ADMSC for the treatment of rats. For characterization, ADMSC were trypsinized and labeled with fluorescein isothiocyanate (FITC)-, phycoerythrin (PE)- or Alexa 647-conjugated primary antibodies. The cells were incubated for 20 minutes at 4 °C in the dark with the following antibodies: CD90-FITC (AbD Serotec, Oxford, UK), CD29-PE (AbD Serotec), CD45-PE (AbD Serotec) and CD11b-PE (AbD Serotec). Matched isotype controls were purchased from Biolegend (San Diego, CA, USA). Flow cytometry analysis of CD90+/CD29+/CD45–/CD11b– cells was performed using a FACScalibur cytometer and CellQuest Pro software (Becton Dickinson, Madrid, Spain). For ADMSC treatment, ADMSC with >95 % viability were administered i.v. The dose, route and time of administration were based on previously reported data [9, 11].

### Proteomics data analysis

For proteomic analysis of cell culture supernants, ADMSC were cultured overnight with a free fetal bovine serum/protein culture medium. After 24 hours, cell supernatants were collected and the protein content was analyzed as follows. Proteins were digested using the filter aided sample preparation (FASP) protocol [12]. Briefly, samples were dissolved in 50 mM Tris–HCl pH 8.5, 4 % SDS and 50 mM DTT, boiled for 10 minutes and then centrifuged. Protein concentration in the supernatant was measured by the Direct Detect® Spectrometer (Millipore, Billerica, MA, USA). Approximately 50 μg of protein was diluted in 8 M urea in 0.1 M Tris–HCl (pH 8.5), and loaded onto 30 kDa centrifugal filter devices (FASP Protein Digestion Kit, Expedeon, Knoxville, TN, USA). The denaturation buffer was replaced by washing three times with UA. Proteins were later alkylated using 50 mM iodoacetamide in UA for 20 minutes in the dark, and the excess alkylation reagents were eliminated by washing three times with UA and three additional times with 50 mM ammonium bicarbonate. Proteins were digested overnight at 37 °C with modified trypsin (Promega, Madison, WI, USA) in 50 mM ammonium bicarbonate at 40:1 protein to trypsin (w/w) ratio. The resulting peptides were eluted by centrifugation with 50 mM ammonium bicarbonate (twice) and 0.5 M sodium chloride. Trifluoroacetic acid (TFA) was added to a final concentration of 1 % and the peptides were finally desalted onto C18 Oasis-HLB cartridges and dried-down for further analysis.

Peptides were loaded into the LC-MS/MS liquid chromatography tandem mass spectrometry system for on-line desalting onto C18 cartridges and analyzing by LC-MS/MS using a C-18 reversed phase nano-column (75 μm internal diameter × 50 cm, 2 μm particle size, Acclaim PepMap RSLC, 100 C18; Thermo Fisher Scientific, Waltham, MA, USA) in a continuous acetonitrile gradient consisting of 0–30 % B in 180 minutes, 50–90 % B in 3 minutes (B = 90 % acetonitrile, 0.5 % formic acid). A flow rate of 200 nL/minute was used to elute peptides from the RP nano-column to an emitter nanospray needle for real time ionization and peptide fragmentation on an Orbitrap Elite mass spectrometer (Thermo Fisher Scientific). An enhanced FT-resolution Fourier- Transform spectrum (resolution = 35,000) followed by the MS/MS spectra from the most intense 15 parent ions were analyzed along the chromatographic run. Dynamic exclusion was set at 30 seconds.

For peptide identification, all spectra were analyzed with Proteome Discoverer (version 1.4.0.29; Thermo Fisher Scientific) using SEQUEST-HT (Thermo Fisher Scientific). For database searching on the Uniprot database containing all sequences from humans (6 March 2013), parameters were selected as follows: trypsin digestion with two maximum missed cleavage sites, precursor and fragment mass tolerances of 600 ppm and 0.02 Da, respectively, carbamidomethyl cysteine as fixed modification and methionine oxidation as dynamic modifications. Peptide identification was validated using the probability ratio method [13] with an additional filtering for precursor mass tolerance of 10 ppm. False discovery rate (FDR) was calculated using inverted databases and the refined method [14]. For the study of the biological functions of identified proteins, gene onthology analysis was performed using the GOrilla (Gene Ontology Enrichment Analysis and Visualization) research tool [15].

### Biodistribution analysis

For identification of donor cells, ADMSC were labeled with DiI (Celltracker CM-DiI, Invitrogen, Barcelona, Spain) prior to administration and stained with CD90, and possible migration and implantation were analyzed using immunofluorescence. Biodistribution of labeled ADMSC with DiI 24 hours after i.v. administration was analyzed using immunofluorescence techniques in both control and treated animals. Cryosections (10 μm thick) of brain, kidney, liver, lung and spleen were counterstained with 4′,6-diamino-2-phenylindole (DAPI) and analyzed by immunofluorescence staining (n = 4 per group).

### Functional evaluation

Functional evaluation was performed in all animals by a blinded observer before surgery and after 1, 3, 7, 14 and 28 days. Motor performance was evaluated using the beam walking and rotarod tests and Rogers’ functional scale. The beam walking test measured the ability of rats to walk along a wooden beam (2.5 × 2.5 × 80 cm). Scores were assigned as follows: 0, traversed the beam with no foot slip; 1, traversed with grasping of the lateral side of the beam; 2, difficulty crawling along the beam but able to traverse; 3, required >10 seconds to traverse the beam because of difficulty with walking; 4, unable to traverse the beam; 5, unable to move the body or any limb on the beam; and 6, unable to stay on the beam for >10 seconds [16]. The rotarod test measured the latency to fall from a rotating cylinder [9]. A variant of Rogers’ functional scale was used to assign scores as follows: 0, no functional deficit; 1, failure to extend forepaw fully; 2, decreased grip of forelimb while tail gently pulled; 3, spontaneous movement in all directions, contralateral circling only if pulled by the tail; 4, circling; 5, walking only when stimulated; 6, unresponsive to stimulation with a depressed level of consciousness; and 7, dead (n = 10 per group) [17].

### In vivo magnetic resonance imaging and tractography

Lesion size was analyzed after 1, 7 and 28 days by magnetic resonance imaging (MRI) using a 7-Tesla horizontal bore magnet (Bruker Pharmascan, Ettlingen, Germany) and T2 maps (RARE 8 T2, 180° flip angle, three averages) as previously described [9]. The lesion area was expressed as a percentage of the contralateral hemisphere, after correcting for brain edema. For tractography, diffusion tensor imaging (DTI) was performed after 1, 7 and 28 days using a spin-echo single-shot echo-planar imaging pulse sequence with the following parameters: TR/TE, 5000/35 ms; signal average, 10; 30 non-collinear diffusion gradients with diffusion weighting of b = 1,000 s/mm^2^ and b = 0 s/mm^2^; and field of view 3.5 × 3.5 cm. A total of 496 slices were evaluated from data acquired in 30 directions. The images were obtained using medInria (Inria, France), a multi-platform medical image processing and visualization software. Zoomed lesion site three-dimensional diffusion tensor images were represented using ParaView 4.1.0 software (Los Álamos National Laboratory, New México, USA) (n = 6 per group, 10 sections per animal) as previously described [10].

### Cell death evaluation

Cell death was analyzed in the infarct zone of at least 10 sections from each animal using TUNEL staining (TdT-FragEL DNA Fragmentation Detection Kit, Oncogene Research Products, San Diego, CA). The number of positive cells was counted in a minimum of 10 different microscopical fields based on their nuclear morphology and dark color using a 40× objective lens and image analysis software (Image-Pro Plus 4.1, Media Cybernetics, Rockville, MD, USA) (n = 6 per group, 10 sections per animal).

### Cell proliferation analysis

Cell proliferation was analyzed using Ki-67 staining (1:100, Chemicon, Temecula, CA, USA) after 7 and 28 days, in 10 sections corresponding to the infarct area of each animal, selected as previously described [18, 19]. The number of positive cells was counted in a minimum of 10 different random microscopic fields using a 40× objective lens and Image-Pro Plus 4.1 software (n = 6 per group, 10 sections per animal).

The differentiation of the proliferating cells was analyzed using co-staining with Ki-67 and NeuN (1:100, Millipore), Olig-2 (1:400, Millipore) and glial fibrillary acidic protein (GFAP; 1:400, Chemicon) followed by goat anti-mouse Alexa Fluor 488 antibody (1:750, Invitrogen). Images were acquired as a confocal maximum projection using a Leica TCS-SPE confocal microscope (Leica Microsystems, Heidelberg, Germany) and the number of double-positive cells was counted in a minimum of 10 different microscopic fields using a 40× objective lens and Image-Pro Plus 4.1 software.

### Immunohistochemical, immunofluorescence and Western blot analyses

Frozen sections were stained using the CryoMyelin Kit (Hitobiotech, Wilmington, USA), which allows sensitive localization and visualization of myelin fibers. The mean intensity of myelin staining in the region of interest (ROI) was quantified using a Nikon Eclipse-Ti inverted microscope and NIS-elements software. The lesion area was studied in detail using immunofluorescence and Western blot analyses. The white matter-associated antibodies used were neurofilament (NF; 1:100, Dako, Glostrup, Denmark), neurite outgrowth inhibitor (NogoA; 1:100, Abcam, Cambridge, UK), myelin basic protein (MBP; 1:100, Abcam) and oligodendrocyte (Olig-2; 1:500, Millipore) followed by goat anti-mouse Alexa Fluor 488 and anti-rabbit Alexa Fluor 594 (1:750, Invitrogen). For Western blot analysis, the units were normalized based on Β-actine (1:400, Sigma-Aldrich). To quantify the expression of white matter-associated markers, the mean fluorescence intensity was evaluated in a minimum of 10 different microscopic fields using a 40× objective lens. The experiments, images and quantification of the samples were performed on the same day using the same microscope configurations, by blinded observers, to eliminate bias due to background normalization (four animals, four sections per animal).

### Statistical analysis

Results were expressed as mean ± standard error of the mean (SEM). Data were compared using the Kruskal-Wallis test followed by the Mann–Whitney test. Values of *p* < 0.05 were considered as statistically significant. The analysis was performed using statistical SPSS 16 and GraphPad software (GraphPad Software Inc, CA, USA).

## Results

### ADMSC characterization, migration and implantation in the injured brain area

**Fig. 1:**
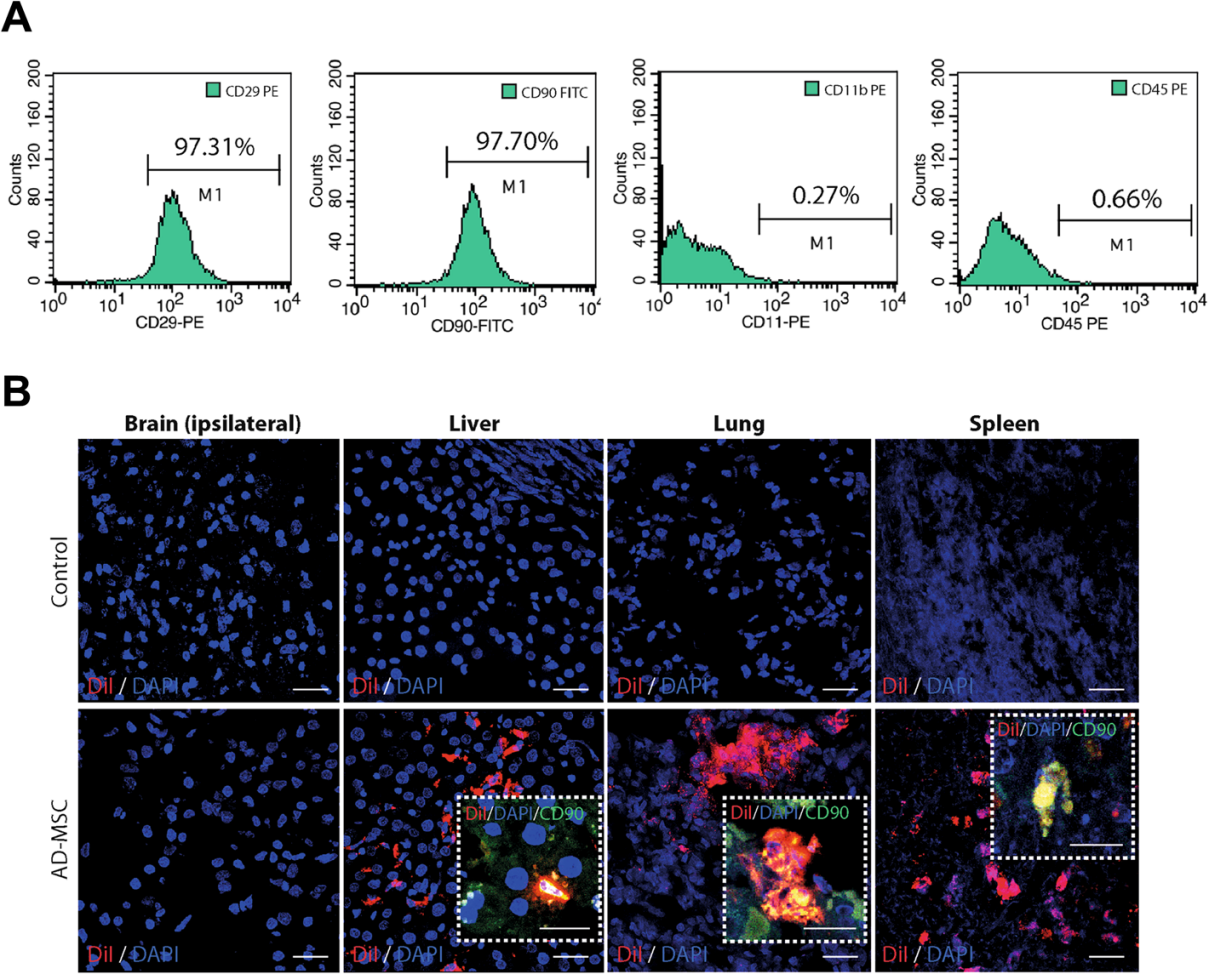
Characterization and biodistribution of ADMSC. **a** ADMSC characterization by flow cytometry. Rat ADMSC were labeled with CD29, CD90, CD11b, and CD45 and analyzed by flow cytometry. Of the ADMSC population, 95 % expressed CD29 and CD90. Additionally, these cells lacked expression (5 % positive) of CD11b, CD45. **b** Migration and implantation in the brain and peripheral organs (liver, lung and spleen) of DiI- and CD90-labelled cells at 24 hours after treatment. *AD-MSC* adipose-derived mesenchymal stem cells, *DAPI* 4′,6-diamino-2-phenylindole, *FITC* fluorescein isothiocyanate, *PE* phycoerythrin

ADMSC showed typical fibroblast-like cell morphology and their phenotype was CD90+/CD29+/CD45–/CD11b– (Fig. 1a).

DiI and CD90 co-labeled cells were not observed in the control group. Migration and implantation in the brain were not observed on immunofluorescence images of the injured brain area after intravenous administration of DiI and CD90 co-labeled cells. However, DiI and CD90 co-labeled cells were observed in peripheral organs such as the liver, lung and spleen (Fig. 1b).

### Effect of ADMSC treatment on functional recovery

**Fig. 2:**
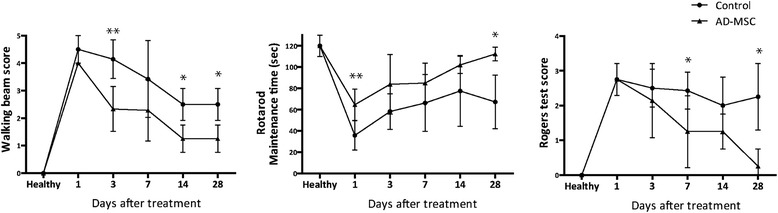
Improved functional outcome after ADMSC administration in subcortical stroke. Beam walking test performance (*left*) was improved at 3 (*p* < 0.01), 14 and 28 days (*p* < 0.05). Rotarod test (*middle*) showed significant differences between the ADMSC group and control animals at 1 (*p* < 0.01) and 28 days (*p* < 0.05). The ADMSC group also showed significantly better scores compared to controls at 7 and 28 days (*p* < 0.05) on the Roger’s test (*right*). Data are shown as mean ± SEM; *p < 0.05, **p < 0.005; n = 10 animals per group. *AD-MSC* adipose-derived mesenchymal stem cells

To assess the potential of ADMSC administration to improve functional recovery after subcortical stroke, motor function was assessed before surgery and after 1, 3, 7, 14 and 28 days using the walking beam, rotarod and Roger’s test. There were significant differences in motor deficit scores between the treatment and control group; the walking beam test performance was significantly better in the treatment group than in the control group after 3 days (*p* < 0.01), 14 days (*p* < 0.05) and 28 days (*p* < 0.05). The Rotarod test was significantly better in the ADMSC treatment group than in the control group after 1 day (*p* < 0.01) and 28 days (*p* < 0.05). The Rogers’ functional scale score was significantly better in the treatment group than in the control group after 7 and 28 days (both *p* < 0.05) (Fig. 2).

### Effect of ADMSC treatment on lesion size and tract connectivity

**Fig. 3:**
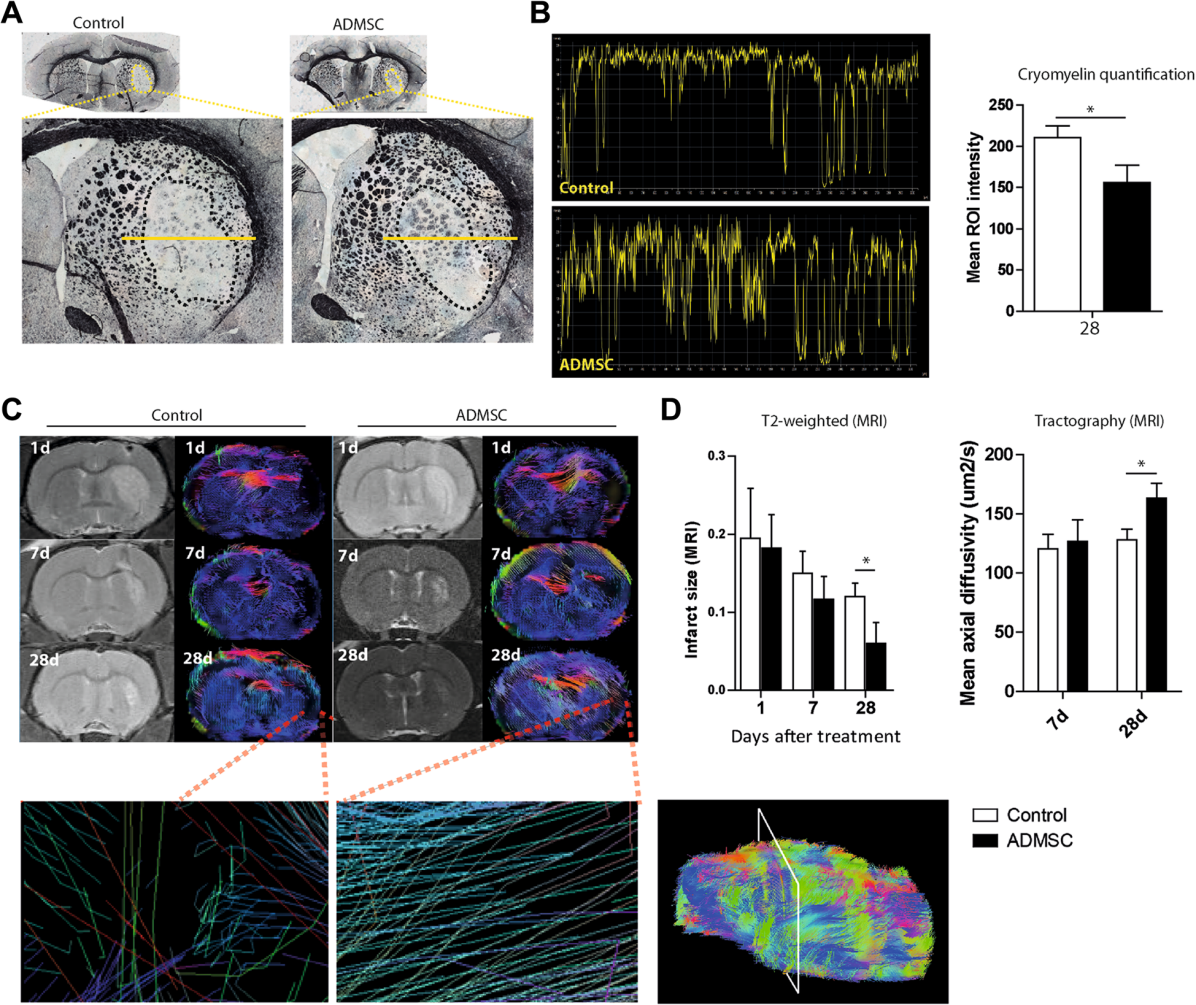
ADMSC treatment reduced infarct size and increased fiber tract and myelin integrity after subcortical white matter damage. **a** Morphological study by CryoMyelin staining identified the zone of the lesion as an area of white matter injury located in the subcortical zone, showing restored myelinated axons in the ADMSC-treated animals. **b** Quantification of mean ROI intensity of the CryoMyelin staining. Stroked line indicates ROI; yellow line indicates a representative longitudinal profile of pixel intensity. **c** Comparative image analysis T2-weighted MRI and tractography at 1, 7 and 28 days showed a progressive reduction in white matter infarct size in controls and treated animals. Detail of tractography image in the lesion is given below showing augmented connectivity of fiber tracts in ADMSC-treated animals at 28 days. **d** Quantitative analysis of MRI images showed that ADMSC therapy reduced lesion size at 28 days compared to the controls (*p* < 0.05). Data are shown as mean ± SEM; *p < 0.05; n = 6 animals, 10 sections each per group. *ADMSC* adipose-derived mesenchymal stem cells, *d* days, *MRI* magnetic resonance image, *ROI* region of interest

MRI analysis showed no significant difference in infarct size between the treatment and control groups after 1 and 7 days. However, the infarct size was significantly smaller in the treatment group than in the control group after 28 days (0.12 ± 0.01 vs. 0.6 ± 0.26, *p* < 0.05) (Fig. 3).

Myelin was stained using the CryoMyelin Kit to identify the area of white matter injury in the subcortical infarct. The mean intensity of staining in the ROI was evaluated in the treatment and control groups, with white indicating absence of myelin and black indicating the presence of myelinated axons. There was higher intensity (indicating absence of myelin) in the control group (210.23 ± 14.30 mean intensity) than in the treatment group (155.71 mean intensity ± 21.23) (Fig. 3).

DTI tractography data showed similar results in axial diffusivity (120.35 ± 12.45 µm^2^/s and 126.78 ± 18.34; *p* > 0.05) in both the control and treated groups, respectively, at 7 days. However, compared with the control rats, 28 days after treatment the ADMSC-treated rats showed significantly improved axial diffusivity (127.98 ± 9.21 and 162.99 ± 13.65 µm^2^/s, respectively; *p* < 0.05) compared to controls. These results suggest that there was a significant improvement in white matter thickness (width, breadth, depth) and restoration of tract connectivity in the ADMSC-treated animals compared with controls at 28 days.

### Effect of acute ADMSC treatment on cell death and brain cell proliferation

**Fig. 4:**
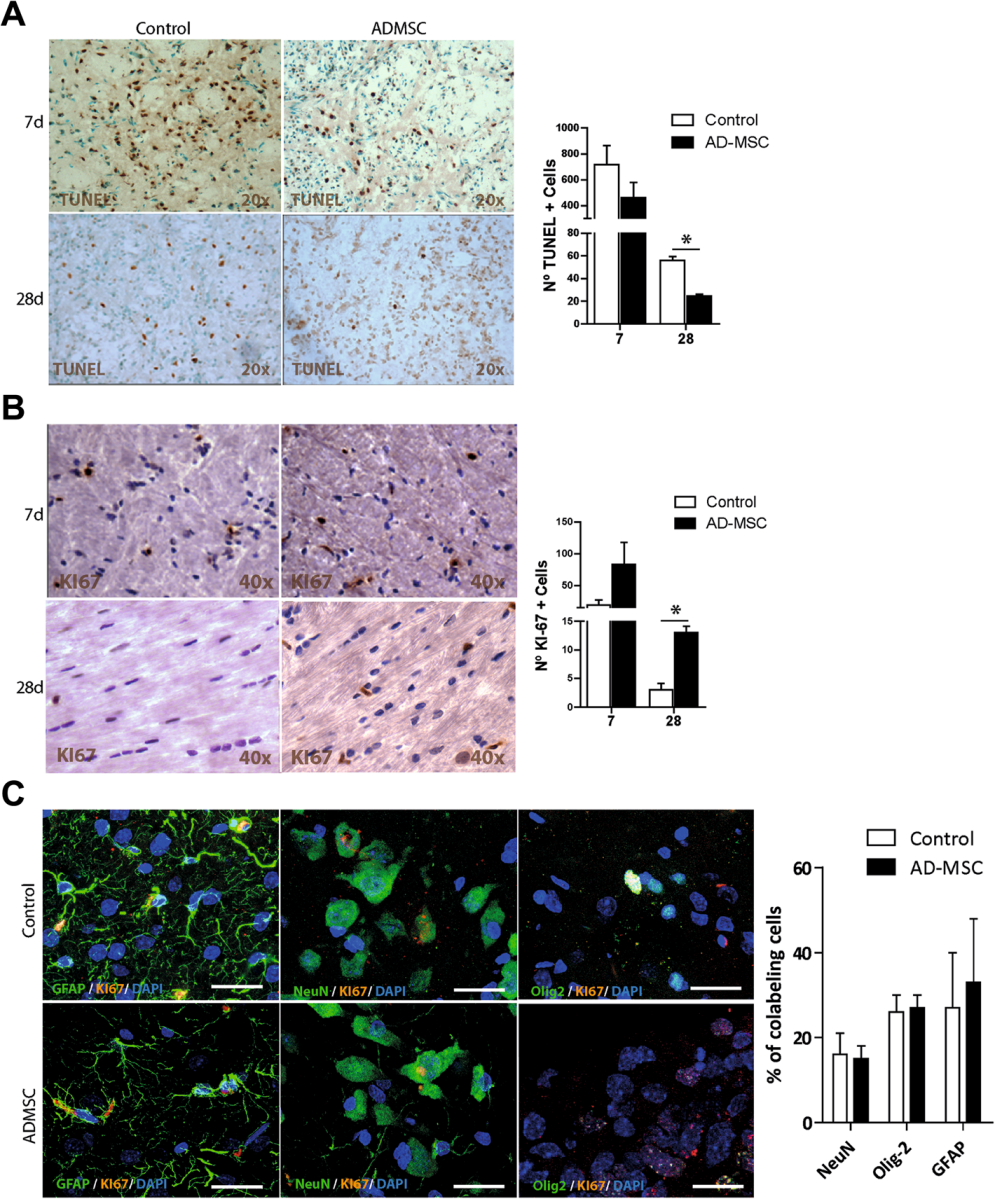
ADMSC administration led to a cell death reduction and improved brain proliferation activity. **a** Quantitative analysis of cell death by TUNEL technique showed a significant reduction in TUNEL-positive cells after ADMSC therapy compared to the control group (*p* < 0.05). **b** At 28 days, Ki-67 staining shows a significant increase in the number of proliferating cells in ADMSC-treated animals compared to the control group (*p* < 0.05). **c** At 28 days after treatment, Ki-67 co-labeling with NeuN, GFAP, and Olig-2 showed different cell type proliferation including oligodendrocytes. Data are shown as mean ± SEM; scale bars = 20 μm; n = 6 animals, 10 sections each per group. *ADMSC* adipose-derived mesenchymal stem cells, *DAPI* 4′,6-diamino-2-phenylindole, *GFAP* glial fibrillary acid protein, *NeuN* neuronal nuclei, *Olig-2* oligodendrocite transcription factor 2

Cell death was analyzed on frozen sections by TUNEL staining after 7 and 28 days. After 7 days, no significant differences were found in TUNEL-positive cells in both the control group (718.5 ± 146.3 cells) and the treatment group (460.33 ± 120.5 cells). After 28 days, there were significantly fewer TUNEL-positive cells in the ischemic area in the treatment group than in the control group (24.5 ± 1.73 vs. 56.0 ± 3.46 cells, *p* < 0.05) (Fig. 4a).

Quantitative analysis of proliferative cells was performed using Ki-67 labeling after 7 and 28 days. After 7 days, there were no significant differences in proliferative cells in both the treatment group (83.5 ± 34.65 cells) and the control group (19.5 ± 7.78 cells) (Fig. 4b). After 28 days, the number of Ki-67-positive cells was significantly higher in the treatment group than in the control group (13 ± 1.15 vs. 3 ± 1.15 cells, *p* < 0.05). The proportions of proliferating cell types observed by co-staining with Ki-67 were not significantly different between the treatment and control groups (NeuN, 15 ± 3 % vs. 16 ± 5 %, Olig-2: 27 ± 3 % vs. 26 ± 4 %; GFAP, 33 ± 15 % vs. 27 ± 13 %) (Fig. 4c). However, although there were no significant differences between the proportions of the cell lines, higher levels of each cell type were found in the ADMSC-treated group.

### Effect of ADMSC treatment on white matter-associated marker expression

**Fig. 5:**
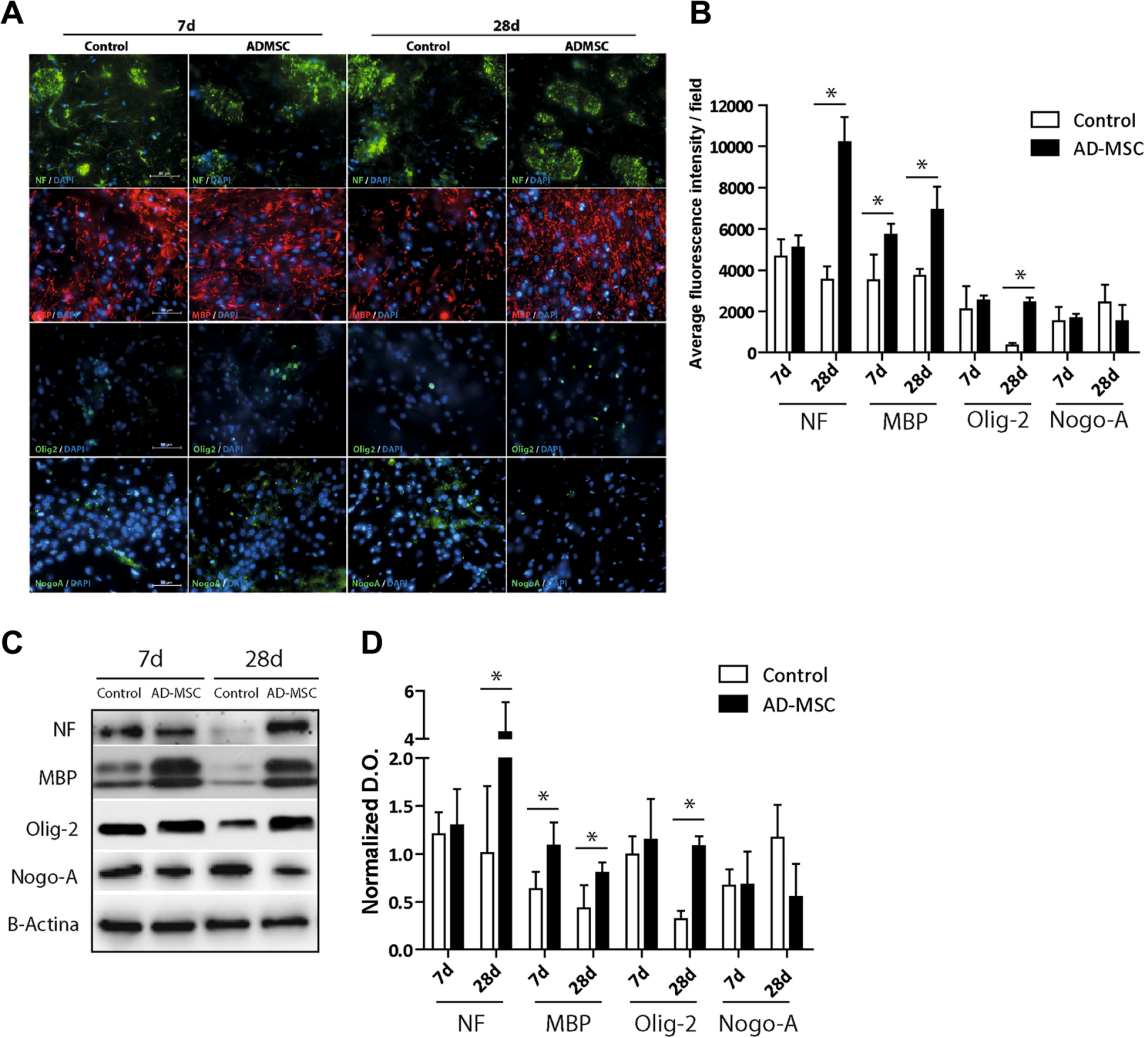
White matter-associated markers are enhanced in striatum after ADMSC therapy in subcortical stroke model. **a** Immunofluorescence images and **b** immunofluorescence quantification of white matter repair-associated markers (NF, MBP, Olig-2 and NogoA) at 7 and 28 days after treatment. **c** Western blot and **d** Western blot quantification showed increased levels of MBP in the treated group compared to controls at both 7 and 28 days (*p* < 0.05), as well as augmented levels of Olig-2, NF (*p* < 0.05) and a trend to decreased levels of NogoA. Data are shown as mean ± SEM; scale bars = 20 μm; *p < 0.05; n = 4 animals, 4 sections each per group. *ADMSC* adipose-derived mesenchymal stem cells, *d* days, *MBP* myelin basic protein, *NF* neurofilament, *Nogo-A* neurite outgrowth inhibitor, *Olig-2* oligodendrocite transcription factor 2

After finding that ADMSC administration had a beneficial effect on functional outcome, we investigated whether the functional outcome was related to the levels of white matter-associated markers. Western blot analysis found that the level of NF (a marker of axonal sprouting) was not significantly different between the treatment and control groups after 7 days. However, the NF level was significantly higher in the treatment group than in the control group after 28 days (4.27 ± 1.26 vs. 1.00 ± 0.69 Arbitrary Units (AU), *p* < 0.001). The MBP level was significantly higher in the treatment group than in the control group after 7 days (1.09 ± 0.23 vs. 0.63 ± 0.18 AU, *p* < 0.05) and 28 days (0.80 ± 0.11 vs. 0.43 ± 0.24 AU, *p* < 0.05). The Olig-2 level was significantly higher in the treatment group than in the control group after 28 days (1.08 ± 0.10 vs. 0.32 ± 0.08 AU, *p* < 0.05). The NogoA level was not significantly different between the treatment and control groups after 7 days (0.68 ± 0.35 vs. 0.67 ± 0.17 AU, *p* > 0.05), and tended to be lower in the treatment group than in the control group after 28 days (0.55 ± 0.35 vs. 1.16 ± 0.34 AU, *p* > 0.05) (Fig. 5). The NF immunofluorescence intensity was significantly higher in the treatment group than in the control group after 28 days (10,020.28 ± 1,231.19 vs. 3,536.21 ± 643.2 average fluorescence intensity, *p* < 0.05). The MBP immunofluorescence intensity was significantly higher in the treatment group than in the control group after 7 days (5,714.61 ± 529.59 vs. 3,529.97 ± 1,222.40 AU, *p* < 0.05) and 28 days (6,920.39 ± 1,134.27 vs. 3,736.34 ± 324.50 AU, *p* < 0.05). The Olig-2 immunofluorescence intensity was significantly higher in the treatment group than in the control group after 28 days (2,439.00 ± 231.12 vs. 353.40 ± 111.12 AU, *p* < 0.05). The NogoA immunofluorescence intensity tended to be lower in the treatment group than in the control group after 28 days (1,534.21 ± 767.32 vs. 2,423.88 ± 876.70 AU, *p* > 0.05).

### Proteomics analysis of the ADMSC in vitro secretome

**Fig. 6:**
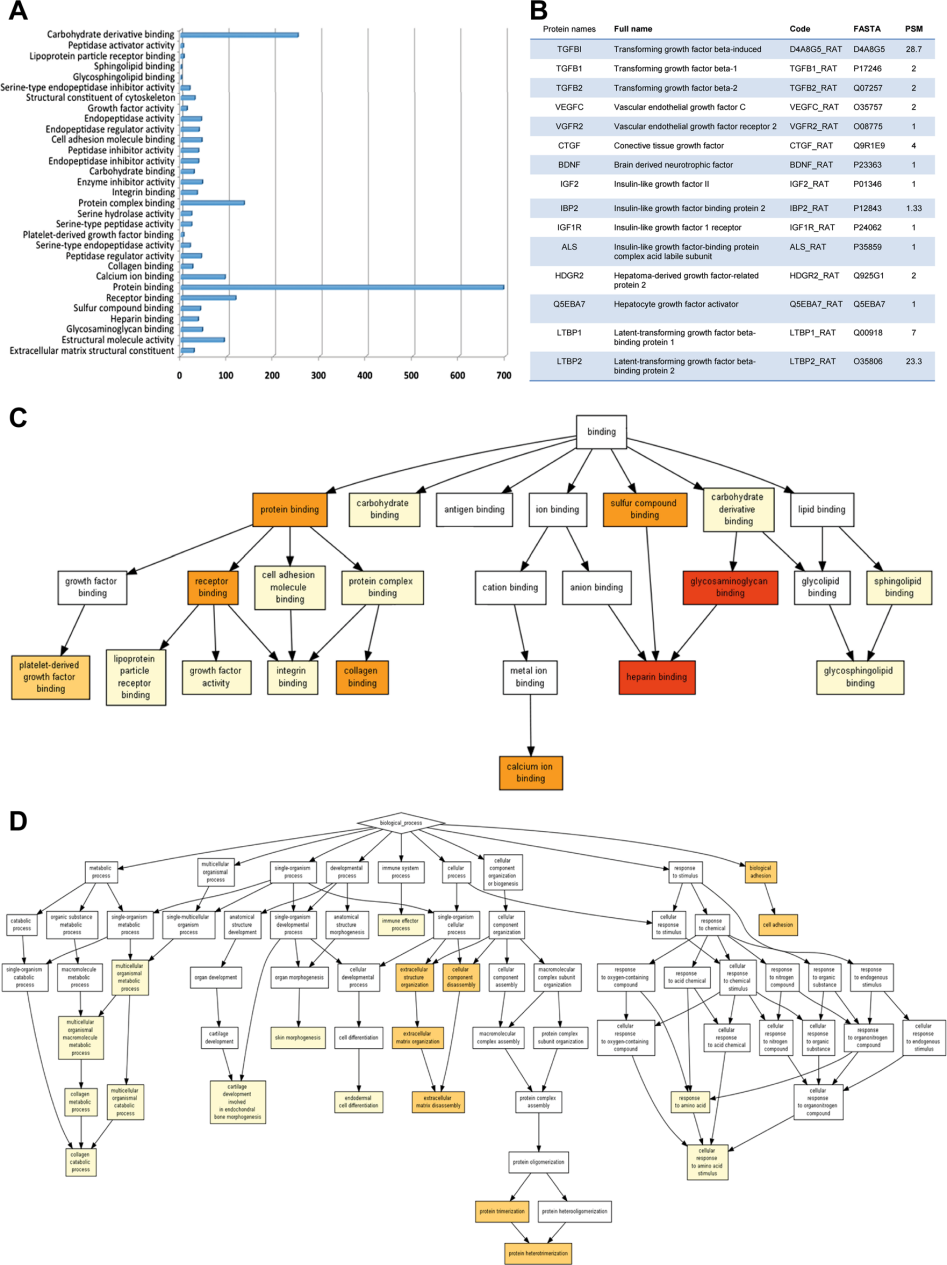
Proteomics analysis of ADMSC secretome reveals multiple biological functions. **a** Gene onthology (GO) analysis of the 2,416 proteins identified by Orbitrap proteomic study. Bars indicate the number of proteins from the total associated with each biological function. **b** Trophic factor-related proteins identified in the proteomic analysis; PSM: peptide- spectrum matches **c** GO functional clustering of proteins associated with binding function and **d** other biological processes

Proteomics analysis of the secretome of cell cultures identified 2,416 proteins in the cell supernatants that are implicated into different cell functions (Fig. 6a), such as protein binding (carbohydrate binding, antigen binding, ion binding, sulfur compound binding, and lipid binding), metabolic processes, single and multicellular organism processes, development, endodermal cell differentiation, skin and cartilage morphogenesis, immune system processes, cellular organization and biogenesis, response to stimulus and biological adhesion processes . All functions in detail are shown in Fig. 6c and d. Full proteomic data is given in Additional file 1. Interestingly, some of these proteins are also implicated in growth factor activity, such as several trophic factors and receptors known to be also involved in brain plasticity. Some of these trophic factors are shown in Fig. 6b.

## Discussion

The results of this study showed that ADMSC administration plays a major role in improving the repair of white matter fiber tracts in an experimental model of subcortical stroke. We found that the treated group had better functional recovery and smaller lesion size than the control group. In addition, animals which received ADMSC treatment showed significantly higher number of proliferating cells (including oligodendrocyte progenitors) and significantly less cell death at the lesion region than animals in the control group. Analysis of fiber tract integrity by tractography and CryoMyelin staining showed that white tract thickness had been recovered in the treatment group. The treated group with ADMSC also had higher levels of white matter-associated markers (NF, MBP and Olig-2) than the control group, suggesting that ADMSC administration induced repair of white matter fiber tracts.

Up to 25 % of ischemic strokes in humans are subcortical or lacunar, which are confined to white matter regions such as the striatum and internal capsule [1]. The high frequency of damage to these areas in stroke patients has motivated the search for useful experimental animal models of subcortical stroke with white matter affectation, as well as effective therapies to enhance the mechanisms underlying repair of damaged white matter (axon and myelin). In this regard, endothelin (one of the most potent known vasoconstrictors) was considered the best candidate to induce this injury with white matter affectation [10, 20, 21].

ADMSC administration is considered an appropriate therapeutic strategy because ADMSC enhance the natural repair processes of the brain after injury. However, the mechanisms underlying these repair processes are still unknown. Our proteomics analysis identified thousands of proteins, many of them not previously associated with stem cell properties or stroke repair; for instance, we identified a number of proteins such as hepatoma-derived growth factor, latent-transforming growth factor beta, and connective tissue growth factor. Other proteins previously implicated in stem cell therapy function such as transforming growth factor-beta, fibroblast growth factor, vascular endothelial growth factor or brain-derived neurotrophic factor were also identified. Moreover, gene onthology analysis identified a number of protein functions not previously associated with stem cell therapy function in stroke recovery. In this regard, protein binding (carbohydrate binding, antigen binding, ion binding, sulfur compound binding, lipid binding), metabolic processes, single and multicelular organism processes, development, endodermal cell differentiation, skin and cartilage morphogenesis, immune system processes, cellular organization and biogenesis, response to stimulus and biological adhesion processes were highly represented. Interestingly, growth factor activity was not the main represented function in the cell secretome, indicating that other functions are also relevant. Our findings suggest that the release of the identified proteins by the administered ADMSC could contribute to improve functional recovery when allocated to peripheral organs (spleen, lung and liver). However, futures studies will be needed in order to understand the complex molecular mechanisms involved in stem cell therapy-mediated stroke recovery.

Various tests have been used to evaluate motor function following brain injury. Previous studies by our group have demonstrated improvement in the functional outcome after ADMSC administration in another experimental animal model of cerebral ischemia [9]. The present study showed that functional recovery at 28 days was significantly improved after ADMSC administration than in the control group after subcortical ischemic stroke.

In the present study, MRI studies showed a significantly smaller infarct size in the treated group than in the control group after 7 and 28 days. Our results are consistent with previous studies that reported a reduction in infarct size after ADMSC administration in another animal model of cerebral ischemia [9]. This reduction in infarct size could be related to the increased tract thickness and axonal projections observed by tractography and CryoMyelin staining. These results agree with previously reported findings that neural progenitor cell treatment in an animal model of cortical ischemia results in white matter reorganization shown by fiber tracking maps derived from DTI and by histological staining [22].

On the other hand, we found that the density of TUNEL-positive cells in the ischemic area peaked after 7 days in both treated and control groups. However, the treated group had less focal damage at 28 days, with significantly lower numbers of TUNEL-positive cells than in the control group. These results are consistent with those of previously reported studies, which found that ADMSC administration inhibited cell death in the infarct area [5, 9].

The central nervous system continuously generates new cells in several specific regions of the adult mammalian brain, and this proliferation has been shown to be enhanced by cell therapy. Ki-67 staining showed large numbers of proliferative cells after 7 days in both the treatment and control groups. In this regard, our results are consistent with a previous study [23]. Furthermore, there was greater cell proliferation after 28 days in the treated group than in the control group. These results provide clear evidence that ADMSC administration induces significant cell proliferation at 28 days after subcortical stroke. Furthermore, analysis of the proliferating cell lines showed similar proportions of co-staining with Ki-67 and NeuN, Olig-2 and GFAP in the treatment and control groups, indicating that almost half of the proliferating cells in both groups were white matter-associated cells (neurons and oligodendrocytes). These findings indicate genesis of new astrocytes and neurons as well as oligodendrocyte progenitors and immature oligodendrocytes after cerebral ischemia. Although the effects of ADMSC on neurogenesis, gliogenesis, synaptogenesis and vasculogenesis after stroke have already been described, the ability of ADMSC to promote oligodendrogenesis after subcortical stroke is a novel finding.

It is known that some brain repair mechanisms are quickly activated after cortical ischemia [24], but there is little information available regarding brain repair mechanisms after subcortical white matter stroke. To increase our understanding of these mechanisms, we investigated the levels of white matter-associated markers (NF, MBP, Olig-2 and NogoA) in both the treatment and control groups. NF levels, a marker of axonal sprouting, were not significantly different between the treated and control groups after 7 days, suggesting that the extent of white matter injury was similar in these groups during the acute phase. However, NF levels were higher in the group treated with ADMSC after 28 days, which could be explained by enhancement of axonal sprouting after ADMSC administration. In addition to axonal growth, restoration of the myelin sheath is important for the repair of white matter. Furthermore, Olig-2 levels were significantly higher in the treated group than in the control group after 28 days. These results support the concept that ADMSC enhance oligodendrogenesis to restore their loss due to ischemic injury. Our findings are supported by those of a recent in vitro study, which found that mesenchymal stem cell-conditioned medium promoted oligodendroglial cell maturation [25]. In addition to formation of mature oligodendrocytes, repair of the myelin sheath by oligodendrocytes is essential for achieving propagation of nerve impulses along axons [26]. The present study analyzed the MBP level as a marker of myelination. There were significantly higher levels of MBP in the treatment group compared to the control group after 7 and 28 days. These higher levels of MBP and Olig-2 are consistent with the increased proliferation observed for all phases of oligodendrocyte progenitors. Our results suggest that ADMSC administration increases oligodendrogenesis after white matter stroke.

Finally, among the myelin-associated proteins, NogoA, myelin-associated glycoprotein and oligodendrocyte myelin-associated protein all share a single receptor complex [27]. The present study did not find a difference in the NogoA level between the treated and control groups after 7 days. However, the NogoA level tended to be lower in the treatment group than in the control group after 28 days. These results may indicate that ADMSC administration can enhance axonal growth and plasticity by reducing the level of NogoA. In addition to indicating axonal sprouting, these findings are in accordance with the tractography and CryoMyelin stain findings that suggest restoration of white tract connectivity in the area of ischemia induced by injection of ET-1.

## Conclusions

The findings of this study support the concept that ADMSC play an important role in enhancing some of the major mechanisms of remyelination. ADMSC administration resulted in a smaller lesion size and less cell death, as well as increased cell proliferation including oligodendrocyte progenitors, and higher levels of white matter-associated markers (NF, MBP and Olig-2) and restoration of white tract connectivity in the infarct area. All these processes may help to explain the improvement in functional outcome after ADMSC administration. Therapies that enhance remyelination may help to prevent the functional deficits resulting from those strokes affecting the white matter.

## Additional file

Additional file 1:
**rADMSC proteomic analysis of cell supernatants.**

## Abbreviations

ADMSC: Adipose tissue-derived mesenchymal stem cells
AU: Arbitrary units
DAPI: 4′,6-diamino-2-phenylindole
DiI: Celltracker CM-DiI
DTI: Diffusion tensor imaging
ET-1: Endothelin-1
FASP: Filter aided sample preparation
FITC: Fluorescein isothiocyanate
GFAP: Glial fibrillary acidic protein
i.v.: Intravenously
LC-MS/MS: Liquid chromatography tandem mass spectrometry
MBP: Myelin basic protein
MRI: Magnetic resonance imaging
NF: Neurofilament
NogoA: Neurite outgrowth inhibitor
Olig-2: Oligodendrocyte transcription factor 2
PE: Phycoerythrin
ROI: Region of interest
SEM: Standard error of the mean
